# Supplementary material for: Low-spin state of Fe in Fe-doped NiOOH electrocatalysts
Source: Nat Commun. 2023 Jun 13;14:3498. doi: 10.1038/s41467-023-38978-5 (PMC10264450; doi:10.1038/s41467-023-38978-5)
Supplement: Supplementary file 1 — Supplementary Information [file 41467_2023_38978_MOESM1_ESM.pdf]

Supplementary Information

S1. Structures used in this work

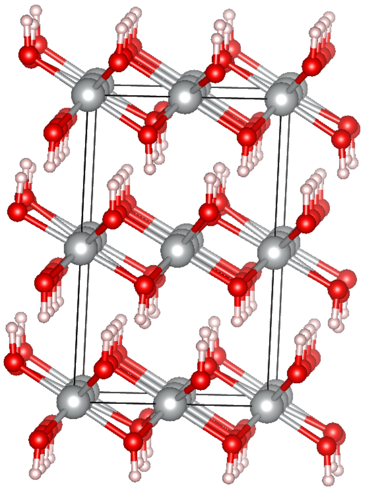

Figure S1: Supercell structure of β-$Ni(OH)_2$ used in this work. The structure is created from reported experimental values [1].

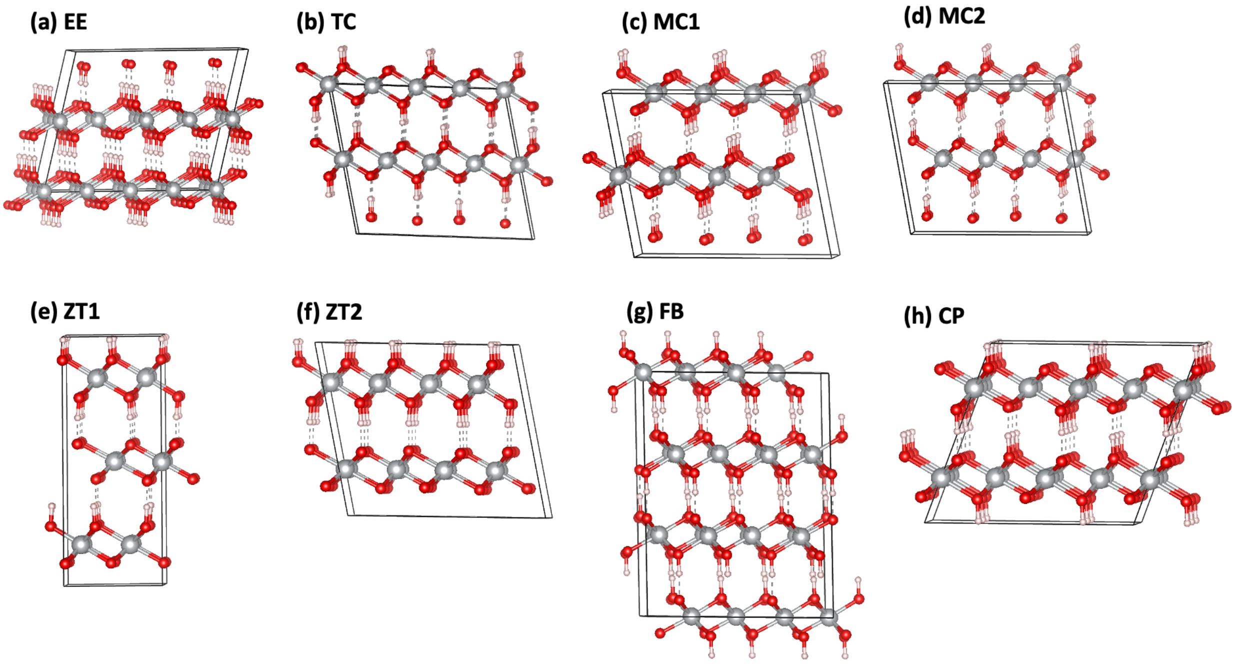

Figure S2: Different supercell structures of β-NiOOH investigated in this work. The names except (h) were adopted from Ref. [2]. CP represents the experimentally observed structure [3].

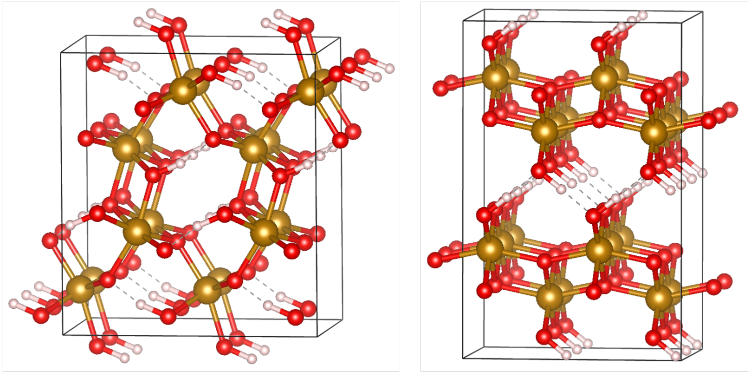

(a) α − FeOOH (geothite)   (b) γ − FeOOH (lepidocrocite)

Figure S3: Structures of α- and γ-FeOOH investigated in this work. The structures are created from the reported experimental data [4].

S2. Relative energies for different structures of β-NiOOH

Table S1: Relative energies for different structures of β-NiOOH computed here with the DFT+U and DFT+U(WF) methods. The last column shows the values computed in Ref. [2]. The corresponding supercell structures are reported in Fig. S2. All energies are given per formula unit and in meV.

	$\Delta E_{\mathrm{DFT}+U}$	$\Delta E_{\mathrm{DFT}+U(\mathrm{WF})}$	$\Delta E_{\mathrm{reported}}$
MC1	9	0	0
MC2	8	0	1
TC	8	0	152
FB	0	0	25
ZT1	92	187	95
ZT2	61	114	22
EE	8	0	5
CP	30	41	

S3. Lattice constants for β-$Ni(OH)_2$ and β-NiOOH

Table S2: Summary of lattice constants of β-NiOOH computed in this work (tw) and reported in previous experimental and theoretical studies. U is the Hubbard parameter value used in the DFT+U method.

**Structure type**	**Methods**	U**(eV)**	a, b, c (Å)	α, β, γ ($\circ$)	Refs.
CP (exp.)			2.442, 2.921, 4.621	90.0, 88.8, 90.0	[3]
KS	DFT(RPBE)		2.810, 2.810, 4.840	90.0, 90.0, 120.0	[5]
CP	DFT+U	5.0	2.559, 2.913, 4.723	90.0, 70.7, 90.0	tw
EE	DFT+U	5.0	2.909, 2.924, 4.883	69.7, 109.8, 119.0	tw
EE	DFT+U	5.5	2.947, 2.993, 4.720	80.2, 87.4, 120.6	[2]
TC	DFT+U	5.0	2.909, 2.959, 4.762	71.4, 105.3, 120.2	tw
TC	DFT+U	5.5	2.926, 2.957, 4.839	80.0, 90.0, 119.7	[2]
MC1	DFT+U	5.0	2.909, 2.959, 4.599	80.2, 87.5, 120.2	tw
MC1	DFT+U	5.5	2.947, 2.993, 4.720	80.2, 87.4, 120.6	[2]
MC2	DFT+U	5.0	2.909, 2.959, 4.883	89.4, 70.2, 120.2	tw
MC2	DFT+U	5.5	2.947, 2.992, 5.004	89.4, 70.3, 120.6	[2]
ZT1	DFT+U	5.0	2.957, 2.928, 4.398	90.2, 90.8, 60.3	tw
ZT1	DFT+U	6.0	2.825, 3.003, 4.446	95.6, 90.0, 61.9	[2]
ZT2	DFT+U	5.0	2.573, 2.905, 4.576	90.0, 101.6, 90.0	tw
ZT2	DFT+U	5.5 & 6	2.572, 2.894, 4.680	90.1, 101.9, 90.1	[2]
FB	DFT+U	5.0	2.950, 2.519, 4.487	87.3, 90.0, 90.0	tw
FB	DFT(RPBE)+U	6.6	2.913, 2.514, 4.721	90.0, 90.0, 90.0	[2]
TC	DFT		2.590, 2.920, 4.620	90.0, 88.8, 90.0	[6]
TC	DFT+U	3.8	2.575, 2.900, 4.670	90.2, 101.9, 90.1	[6]
TC	DFT+U	5.5	2.579, 2.898, 4.669	90.2, 101.9, 90.1	[6]
TC	DFT+U	6.0	2.572, 2.895, 4.695	90.1, 101.8, 90.1	[6]
TC	DFT(optPBE)		2.576, 2.913, 4.669	91.1, 101.5, 90.5	[6]
TC	DFT(optB86)		2.546, 2.877, 4.635	91.1, 101.5, 90.5	[6]
TC	DFT(optB88)		2.562, 2.896, 4.654	91.1, 101.5, 90.5	[6]
TC	DFT(PBE0)		2.555, 2.880, 4.620	88.4, 100.9, 89.5	[6]
TC	DFT(HSE06)		2.560, 2.870, 4.620	88.7, 100.7, 89.6	[6]

Table S3: Lattice constants of β-$Ni(OH)_2$ reported in literature and computed here (tw).

Methods	a, b (Å)	c (Å)	Refs.
DFT+U	3.196	4.497	tw
exp.	3.070	4.610	[7]
exp.	3.114	4.167	[8]
exp.	3.126	4.605	[9]
exp.	2.810	4.840	[10]
exp.	3.127	4.606	[5]
exp.	3.180	4.610	[11]
exp.	3.130	4.630	[12]

S4. Structural parameters of Fe-doped β-NiOOH

Table S4: Computed volumes of β-NiOOH supercell: pure and doped with 1 Fe in high-spin (HS) and low-spin (LS).

System	Volume (Å^3)
β-NiOOH	532.9
Fe(HS):NiOOH	536.5
Fe(LS):NiOOH	531.8

Table S5: The range of computed bond lengths of Ni-O and Fe-O in β-NiOOH and γ-NiOOH. The structure of γ-NiOOH was adopted from Ref. [13].

System	Ni-O (Å)	Fe-O (Å)
Fe(HS):β-NiOOH	1.91-1.92	1.98-2.03
Fe(LS):β-NiOOH	1.88-1.95	1.90-1.92
Fe(HS):γ-NiOOH	1.86-1.99	1.99-2.07
Fe(LS):γ-NiOOH	1.86-1.90	1.84-1.94
exp. [13]	1.90-1.92	1.90-1.94

S5. Estimation of the spin transition energy

All energies for estimating the spin transition of Fe in Fe:NiOOH were computed with the DFT+U(WF) method. The energy differences between configurations with high-spin (HS) and low-spin (LS) Fe species are reported in Table.S6.

Table S6: Energy differences between β-NiOOH dopped with high-spin and low-spin Fe.

Level of Fe doping	Fe content (%)	$\Delta E_{\text{LS-HS}}$ (kJ/mol)
1 Fe	6.25	-39.4
16 Fe	100	105.0

Table S7: Oxidation states of metal cations in *OH active site on Fe-doped NiOOH(0001) surface at different OER steps represented by the R2 reaction pathway. The $+n$ represents oxidation state of cation, the number in parentheses is the total occupation of d orbital. The bold font indicates which metal cation is electrochemically active (exchanges electrons).

		Ni(1)	Ni(2)	Fe
Fe-HS	*OH	+3 (7.0)	+3 (7.0)	+3 (5.0)
	*O	**+4 (6.0)**	+3 (7.0)	+3 (5.0)
	*OOH	+3 (7.0)	+3 (7.0)	+3 (5.0)
Fe-LS	*OH	+3 (7.0)	+3 (7.0)	+3 (5.0)
	*O	+3 (7.0)	+3 (7.0)	**+4 (4.0)**
	*OOH	+3 (7.0)	+3 (7.0)	+3 (5.0)

S6. Crystal Structure Data

Here we list all key computed bulk and surface structures (lattice vectors and atomic coordinates in Å).

Bulk β-NiOOH

Supercell lattice vectors

5.47397615, 0.00000000, -1.96975597

-4.56896877, 9.96805789, 4.06477143

0.00000000, 0.00000000, 9.76550200

Atomic positions

Ni 0.00000091 0.00000997 4.83591775

Ni 1.59474498 2.49200450 4.86721867

Ni 0.90500010 9.96803795 11.81364083

Ni 1.59473950 2.49200450 9.74994234

Ni 3.18949086 4.98401898 4.89853354

Ni -0.68972665 7.47605339 6.89960463

Ni -2.28448257 4.98404888 11.75101749

Ni -0.68974307 7.47605339 11.78231271

Ni 2.73698898 0.00000997 3.85103976

Ni 4.33173305 2.49200450 3.88234068

Ni -1.83198798 9.96803795 12.79851882

Ni 4.33172758 2.49200450 8.76506436

Ni 0.45250278 4.98401898 5.88341152

Ni -3.42672020 7.47605339 7.88448458

Ni 0.45250003 4.98404888 10.76614148

Ni -3.42673114 7.47605339 12.76720046

H 0.91658919 1.15362328 2.49148325

H -0.46409007 3.83041564 3.34508932

H 0.91716301 1.15367312 7.37415706

H -0.46463923 3.83033589 8.22791114

H -1.36788791 6.13767216 4.52386142

H -2.74860454 8.81441468 5.37747860

H -1.36735788 6.13772200 9.40652169

H -2.74911905 8.81435487 10.26028303

H 3.65413647 1.15369305 1.50653817

H 2.27233970 3.83035583 2.36026099

H 3.65359368 1.15362328 6.38932106

H 2.27290167 3.83039570 7.24296594

H 1.36964752 6.13773197 3.53891819

H -0.01214102 8.81436484 4.39265031

H 1.36911021 6.13766219 8.42169911

H -0.01157815 8.81441468 9.27532806

O 0.47020594 1.43510129 1.10004577

O -0.72579722 3.98691415 2.34543516

O 1.17830363 0.99714470 3.49112986

O -0.01771134 3.54888778 4.73648703

O 0.47025800 1.43501158 5.98289296

O -0.72607907 3.98682443 7.22819744

O 1.17858004 0.99717461 8.37385702

O -0.01777621 3.54901736 9.61915613

O -1.81426297 6.41918008 3.13243802

O -3.01029070 8.97090322 4.37781447

O -1.10617071 5.98116368 5.52349978

O -2.30218844 8.53293666 6.76887497

O -1.81421086 6.41903056 8.01527067

O -3.01055069 8.97087332 9.26058340

O -1.10591167 5.98124342 10.40623552

O -2.30226150 8.53303634 11.65152998

O 3.20725612 1.43500161 0.11523841

O 2.01090625 3.98685434 1.36054741

O 3.91556445 0.99719454 2.50624396

O 2.71922738 3.54900740 3.75152891

O 3.20719672 1.43513120 4.99794460

O 2.01118176 3.98687427 6.24331154

O 3.91528898 0.99711480 7.38896730

O 2.71927307 3.54890771 8.63434668

O 0.92278083 6.41907043 2.14762077

O -0.27357081 8.97084341 3.39293057

O 1.63109101 5.98122349 4.53861597

O 0.43475028 8.53305628 5.78392661

O 0.92273333 6.41915018 7.03032034

O -0.27328987 8.97092316 8.27568773

O 1.63080732 5.98117365 9.42135930

O 0.43479326 8.53292669 10.66671856

Bulk β-NiOOH doped with 1 Fe(HS)

Supercell lattice vectors

5.49028625, 0.00000000, -1.94367595

-4.59515824, 10.00536268, 4.07087979

0.00000000, 0.00000000, 9.76593400

Atomic positions

Fe 0.89507591 10.00533266 6.96331524

Ni -1.15526379 2.51894010 5.86797024

Ni -1.85007820 10.00533266 7.93513757

Ni 1.60165819 2.52565370 4.90720625

Ni 3.19263856 5.00265132 4.92785188

Ni 2.05026564 7.48636254 5.93142510

Ni 0.44749543 5.00265132 5.89968985

Ni -0.70665634 7.47964894 6.89216957

Ni 0.89506314 10.00531265 11.84628187

Ni 4.34019280 2.50315164 8.79548016

Ni -1.85007450 10.00531265 12.81811790

Ni 1.59371584 2.50254131 9.76735804

Ni 3.19263677 5.00263131 9.81081462

Ni -3.44520002 7.50211098 12.76985239

Ni 0.44749364 5.00263131 10.78265260

Ni -0.69872765 7.50273132 11.79796881

H 0.90067784 1.16877644 2.49651373

H -0.47019215 3.85497619 3.35360841

H 3.57771254 1.23246057 1.53169673

H 2.27192287 3.84266960 2.37701385

H -1.38032444 6.15361822 4.53036034

H -2.76588481 8.85370541 5.38626267

H 1.37460471 6.15014636 3.55642668

H -0.03004287 8.78140666 4.39979277

H 0.92505940 1.22387597 7.39963155

H -0.47961474 3.85514628 8.24296066

H 3.66089036 1.15157722 6.41314601

H 2.27532450 3.85166442 7.26902098

H -1.37692831 6.16261304 9.42235965

H -2.68269512 8.77283207 10.26771996

H 1.36519221 6.15030644 8.44576314

H -0.00568238 8.83651620 9.30289119

O 0.46480829 1.44056211 1.11526691

O -0.73930926 4.00330569 2.35493300

O 1.15724756 1.00127666 3.50430407

O -0.01252991 3.58001882 4.74999895

O 3.20467117 1.44720567 0.12613922

O 2.00586167 4.00312560 1.38097080

O 3.86261121 1.07139425 2.52098043

O 2.72891885 3.55719659 3.77703850

O -1.83131226 6.44003173 3.13880985

O -3.04117778 9.00443620 4.38579952

O -1.12062414 5.98969036 5.52868612

O -2.30166440 8.57184434 6.76817597

O 0.91392646 6.44257309 2.16211921

O -0.29902386 8.99741244 3.40146772

O 1.63743320 5.99099105 4.55314086

O 0.42341597 8.48100565 5.73797781

O 0.47159775 1.52430700 6.06142554

O -0.74243684 4.01431159 7.24623689

O 1.19403311 1.00785019 8.39794453

O -0.01893918 3.56268953 9.63724222

O 3.19666625 1.43345831 5.03121938

O 2.01562050 4.01561229 6.27069164

O 3.93618154 1.00082642 7.41360490

O 2.72630503 3.56523089 8.66056917

O -1.83391790 6.44809605 8.02233518

O -2.96758919 8.93388839 9.27843219

O -1.11087349 6.00214703 10.41840251

O -2.30966103 8.55806696 11.67327515

O 0.90752537 6.42527382 7.04937668

O -0.26224111 9.00401598 8.29509697

O 1.63430293 6.00196694 9.44444815

O 0.43019636 8.56471052 10.68412987

Bulk β-NiOOH doped with 1 Fe(LS)

Supercell lattice vectors

5.48378103, 0.00000000, -1.93089564

-4.60438397, 9.95188179, 4.07902177

0.00000000, 0.00000000, 9.74645100

Atomic positions

Fe 0.87933322 9.95184198 6.97457157

Ni 4.32443095 2.48299451 3.92442609

Ni -1.86255730 9.95184198 7.94001939

Ni 1.58103993 2.48556209 4.89596806

Ni 3.18153069 4.97590109 4.93505875

Ni -3.44516069 7.46881762 7.87659306

Ni 0.43964017 4.97590109 5.90051632

Ni -0.70177515 7.46625003 6.90505302

Ni 0.87933322 9.95184198 11.84779707

Ni 4.33179994 2.48571137 8.79689744

Ni -1.86255730 9.95184198 12.81324489

Ni 1.59048466 2.48508440 9.76750437

Ni 3.18152520 4.97590109 9.80829593

Ni -3.45253056 7.46609080 12.75057057

Ni 0.43963469 4.97590109 10.77374375

Ni -0.71121528 7.46671777 11.77996364

H 0.79907038 1.11402360 2.55741243

H -0.47662343 3.82247799 3.36162700

H 3.61234174 1.14472515 1.55628451

H 2.26634265 3.82620994 2.39985073

H -1.38061599 6.12792097 4.53081330

H -2.77077717 8.81106772 5.39277651

H 1.36028205 6.12947346 3.56663441

H -0.03319776 8.78158029 4.42078459

H 0.91247263 1.17022188 7.38023049

H -0.48101180 3.82233866 8.23439450

H 3.65004655 1.14073445 6.40824049

H 2.25989173 3.82389115 7.27021367

H -1.38706779 6.12559223 9.40117409

H -2.73306688 8.80707702 10.24473057

H 1.35589829 6.12932418 8.43939782

H 0.08019439 8.83778852 9.24360866

O 0.45359041 1.42187511 1.12472133

O -0.74347402 3.97949878 2.36316420

O 1.07325712 0.95891357 3.55122016

O -0.02009124 3.54063074 4.74865535

O 3.19957810 1.42691076 0.15120910

O 1.99905353 3.98081243 1.39928821

O 3.88887961 0.97818041 2.54623573

O 2.71970203 3.54054118 3.78166270

O -1.84122643 6.40823562 3.14080741

O -3.04834472 8.95435491 4.39787289

O -1.11602172 5.97267161 5.52851004

O -2.29537785 8.53837621 6.78598786

O 0.89815156 6.41008667 2.17528819

O -0.30958839 8.94730898 3.42445921

O 1.62890207 5.97534867 4.56291846

O 0.43721488 8.52233377 5.79775675

O 0.44204989 1.42947835 6.00325459

O -0.74962173 3.97645350 7.23809469

O 1.18885777 1.00449319 8.37655779

O -0.01887670 3.54171550 9.62572689

O 3.17464262 1.41343591 5.01503323

O 1.99529747 3.97914051 6.27251694

O 3.92761958 0.99744726 7.40314219

O 2.72050130 3.54356655 8.66020766

O -1.84042629 6.41127095 8.01936427

O -3.00960935 8.97363171 9.25478343

O -1.11977867 5.97098974 10.40173661

O -2.32030872 8.52489141 11.64980791

O 0.89936699 6.41118138 7.05237162

O -0.19399235 8.99289855 8.24980092

O 1.62274800 5.97229344 9.43785848

O 0.42567897 8.52992706 10.67629568

Bulk FeOOH with β-NiOOH structure (Fe(HS))

Supercell lattice vectors

5.82849122, 0.00000000, -1.96779413

-5.16625560, 10.45061710, 3.93233499

0.00000000, 0.00000000, 9.61826900

Atomic positions

Fe 0.66216885 10.45057530 6.72751183

Fe 4.53686055 2.61261247 3.77826059

Fe -2.25207676 10.45057530 7.71140890

Fe 1.62261495 2.61261247 4.76215766

Fe 3.24529665 5.22526675 4.76134434

Fe 1.95373275 7.83792103 5.74442809

Fe 0.33105105 5.22526675 5.74524141

Fe -0.96051286 7.83792103 6.72832515

Fe 0.66216885 10.45057530 11.53664633

Fe 4.53686055 2.61261247 8.58739509

Fe -2.25207676 10.45057530 12.52054340

Fe 1.62261495 2.61261247 9.57129216

Fe 3.24529665 5.22526675 9.57047884

Fe 1.95373275 7.83792103 10.55356259

Fe 0.33105105 5.22526675 10.55437591

Fe -0.96051286 7.83792103 11.53745965

H 0.81215022 1.30576280 2.35950590

H -0.48117177 3.91946214 3.33956433

H 3.72639583 1.30576280 1.37559922

H 2.43307384 3.91946214 2.35567688

H -1.77097758 6.53107136 4.32567339

H -3.06429957 9.14477070 5.30573183

H 1.14326803 6.53107136 3.34176671

H -0.15005396 9.14477070 4.32184438

H 0.81215022 1.30576280 7.16862116

H -0.48117177 3.91946214 8.14871807

H 3.72639583 1.30576280 6.18474333

H 2.43307384 3.91946214 7.16481138

H -1.77097758 6.53107136 9.13478866

H -3.06429957 9.14477070 10.11488556

H 1.14326803 6.53107136 8.15091083

H -0.15005396 9.14477070 9.13097888

O 0.43189113 1.54229162 1.23417440

O -0.85966456 4.15525942 2.21798468

O 1.19064302 1.06996553 3.48109517

O -0.10091267 3.68293333 4.46491507

O 3.34614257 1.54229162 0.25027537

O 2.05458105 4.15525942 1.23408761

O 4.10488863 1.06996553 2.49720772

O 2.81333293 3.68293333 3.48100838

O -2.15123667 6.76760017 3.20034190

O -3.44279236 9.38056797 4.18415217

O -1.39248478 6.29527408 5.44726266

O -2.68404047 8.90824188 6.43108256

O 0.76301476 6.76760017 2.21644286

O -0.52854675 9.38056797 3.20025511

O 1.52176082 6.29527408 4.46337522

O 0.23020513 8.90824188 5.44717588

O 0.43189696 1.54229162 6.04330693

O -0.85966456 4.15525942 7.02711918

O 1.19064302 1.06996553 8.29023929

O -0.10091267 3.68293333 9.27403995

O 3.34613674 1.54229162 5.05941183

O 2.05458688 4.15525942 6.04322015

O 4.10488863 1.06996553 7.30633260

O 2.81333293 3.68293333 8.29015250

O -2.15123084 6.76760017 8.00947443

O -3.44279236 9.38056797 8.99328667

O -1.39248478 6.29527408 10.25640678

O -2.68404047 8.90824188 11.24020744

O 0.76300894 6.76760017 7.02557933

O -0.52854093 9.38056797 8.00938764

O 1.52176082 6.29527408 9.27250010

O 0.23020513 8.90824188 10.25632000

Bulk FeOOH with β-NiOOH structure (Fe(LS))

Supercell lattice vectors

5.35710358, 0.00000000, -1.65013579

-4.46912726, 9.73731816, 3.29957319

0.00000000, 0.00000000, 9.33253700

Atomic positions

Fe 0.88792009 9.73728894 6.27092127

Fe 4.23977805 2.43426138 3.79622983

Fe -1.79064508 9.73725973 7.09597819

Fe 1.56122626 2.43426138 4.62129773

Fe 3.12246140 4.86862013 4.62112096

Fe 2.00517689 7.30297888 5.44602086

Fe 0.44393282 4.86863960 5.44619489

Fe -0.67338025 7.30297888 6.27109041

Fe 0.88787723 9.73728894 10.93719364

Fe 4.23974499 2.43431006 8.46249947

Fe -1.79062634 9.73728894 11.76224669

Fe 1.56118338 2.43431980 9.28758164

Fe 3.12250426 4.86862013 9.28739493

Fe 2.00520995 7.30293019 10.11228823

Fe 0.44390872 4.86861039 10.11246504

Fe -0.67334184 7.30293019 10.93735612

H 0.78051456 1.21696029 2.28859279

H -0.33663170 3.65160142 3.11288059

H 3.45907170 1.21696029 1.46354191

H 2.34189061 3.65163063 2.28771555

H -1.45400352 6.08559015 3.93847158

H -2.57129090 8.52032866 4.76243944

H 1.22453308 6.08559989 3.11337295

H 0.10729573 8.52029945 3.93746703

H 0.78056546 1.21693107 6.95497983

H -0.33670762 3.65165011 7.77895811

H 3.45908778 1.21696029 6.12982412

H 2.34185400 3.65164037 6.95392259

H -1.45406426 6.08562910 8.60460782

H -2.57120963 8.52027997 9.42890660

H 1.22452237 6.08559989 7.77963542

H 0.10733234 8.52028971 8.60380633

O 0.45645206 1.42502730 1.14717485

O -0.66067715 3.85950290 1.97215264

O 1.10455018 1.00906854 3.42925102

O -0.01259333 3.44356362 4.25423707

O 3.13500385 1.42502730 0.32210695

O 2.01787642 3.85952237 1.14706938

O 3.78310108 1.00905881 2.60418148

O 2.66595310 3.44356362 3.42916149

O -1.77807137 6.29365717 2.79695263

O -2.89525596 8.72817171 3.62191784

O -1.13002864 5.87773736 5.07905355

O -2.24719893 8.31223243 5.90401985

O 0.90047506 6.29365717 1.97187705

O -0.21670059 8.72815224 2.79686366

O 1.54852940 5.87774710 4.25397632

O 0.43135911 8.31224217 5.07896128

O 0.45649314 1.42500783 5.81344552

O -0.66068250 3.85950290 6.63840413

O 1.10453946 1.00906854 8.09554149

O -0.01262994 3.44357335 8.92050010

O 3.13503958 1.42500783 4.98836060

O 2.01786393 3.85950290 5.81334722

O 3.78308320 1.00909776 7.27046318

O 2.66592185 3.44357335 8.09544154

O -1.77811781 6.29367664 7.46322995

O -2.89524525 8.72817171 8.28821104

O -1.13001345 5.87772762 9.74530612

O -2.24715696 8.31222269 10.57029216

O 0.90044023 6.29368638 6.63816205

O -0.21668721 8.72818145 7.46313381

O 1.54853656 5.87770815 8.92024426

O 0.43139483 8.31222269 9.74522427

***O surface with Fe(HS)**

Supercell lattice vectors

5.89489800, 0.00000000, 0.00000000

-3.04229787, 5.15339025, 0.00000000

0.00000000, 0.00000000, 26.99998900

Atomic positions

Fe -0.90464552 2.20531090 5.15942790

Fe 2.32409049 4.07416727 14.27419218

Ni 1.94944404 2.20013175 5.28287185

Ni 0.62964172 3.16112050 9.73157904

Ni 3.57906175 3.15347802 9.70727905

Ni -0.70679516 4.06825633 14.15242223

Ni -2.50131022 4.79413196 5.27973985

Ni 0.45323788 4.78411377 5.28327685

Ni 2.15510861 0.58090561 9.71983404

Ni 5.09599314 0.58029236 9.71629704

Ni 0.82908498 1.47971871 14.16449123

Ni 3.77895707 1.49475630 14.15636423

H 3.58190577 1.27597943 7.35028801

H 0.67156690 1.48535137 7.40566498

H -1.02205985 3.95261425 7.62358189

H 1.90233402 3.91451008 7.64013289

H -2.29201382 4.88247653 12.02185310

H 0.62261439 4.96131824 12.11098007

H -0.68125488 2.39408990 11.80390919

H 2.30352405 2.34313833 11.80647419

H 0.67472284 3.19475668 16.24554238

H 3.11946409 2.94828033 3.25031268

O 2.32134103 0.59167620 15.04874087

O -0.73255951 2.35556830 10.75587762

O 2.22624226 2.36329839 10.75528362

O 3.82776086 3.11140059 15.13265683

O 0.80712089 3.23230428 15.27548678

O 3.33050549 2.95814907 4.20948729

O 0.45737329 3.23504588 4.24345327

O 4.97366987 0.39811486 4.34794323

O 2.02269189 0.52531599 4.40885520

O 0.70779794 1.47777073 8.82432540

O 3.64984509 1.44297504 8.82996840

O -0.65996253 2.38512300 13.26582360

O 2.29001359 2.28945546 13.25772360

O 3.53332231 1.27169181 6.30241843

O 0.58174384 1.44641235 6.33884142

O 1.91016208 3.89771518 6.16315249

O -1.03763679 3.97917998 6.18299748

O -0.95481979 3.94845546 8.67914646

O -2.38864769 4.85807007 10.57781269

O 0.55585055 4.85200453 10.64196466

O 1.98090704 3.95967439 8.68373646

O 5.25962505 0.72931294 15.08886285

O -2.21635570 4.90281180 13.06610468

O 0.72837957 4.96281788 13.17391563

***O surface with Fe(LS)**

Supercell lattice vectors

5.89489800, 0.00000000, 0.00000000

-3.04229787, 5.15339025, 0.00000000

0.00000000, 0.00000000, 26.99998900

Atomic positions

Fe -0.90667214 2.20732588 5.30096184

Fe 2.24325062 4.07641930 14.13800424

Ni 1.98403326 2.20568710 5.30015184

Ni 0.61275613 3.15373569 9.73484603

Ni 3.56281149 3.14648487 9.70358005

Ni -0.76096920 4.07834151 14.13865224

Ni -2.46711473 4.80586623 5.30169084

Ni 0.48058545 4.77988799 5.30889984

Ni 2.13896994 0.57313430 9.72023904

Ni 5.07982291 0.57279417 9.71829504

Ni 0.78284921 1.47983208 14.13695124

Ni 3.72618222 1.50488787 14.13144324

H 3.60162067 1.32021098 7.36292400

H 0.56976183 1.51098433 7.40928298

H -0.99786778 3.92654325 7.63945789

H 1.91755852 3.89043860 7.63945789

H -2.35604905 4.82508837 12.01866710

H 0.66966676 4.93285092 12.09672407

H -0.71229826 2.40486564 11.80750019

H 2.27148871 2.36457128 11.78930220

H 0.87514575 3.38616390 16.15090742

H 3.50466240 2.78642265 3.32035065

O 2.25411909 0.60558004 15.00408289

O -0.76164439 2.36370551 10.76308662

O 2.20225686 2.34595208 10.74245862

O 3.66930906 3.18393971 15.04874087

O 0.81285977 3.30275628 15.17464182

O 3.50363050 2.94177675 4.29064925

O 0.45453147 3.13065881 4.36481822

O 5.00018136 0.50561458 4.43531519

O 2.03606794 0.53297393 4.44187619

O 0.64681932 1.47594643 8.82932040

O 3.64948817 1.44747395 8.80763941

O -0.69586679 2.39032277 13.27575959

O 2.25018879 2.35313075 13.25199960

O 3.54480440 1.30954861 6.29450744

O 0.52552746 1.46401118 6.34343142

O 1.95997947 3.89309259 6.15756349

O -0.99128652 3.92832632 6.18529248

O -0.95843376 3.95216075 8.69051346

O -2.41997567 4.83642583 10.59277068

O 0.58304659 4.83983738 10.64817466

O 1.97549360 3.93767972 8.67957846

O 5.21981676 0.62602354 15.00481189

O -2.27687618 4.85527693 13.07396167

O 0.73279630 4.94848630 13.17167463

***O surface for pure NiOOH**

Supercell lattice vectors

5.89493800, 0.00000000, 0.00000000

-3.04231207, 5.15341903, 0.00000000

0.00000000, 0.00000000, 26.99998700

Atomic positions

Ni -1.03228878 2.23397623 5.29172745

Ni 1.86051574 2.22232435 5.28087346

Ni 0.62950574 3.14719300 9.73508831

Ni 3.57778108 3.14021527 9.70600933

Ni -0.65512181 4.05028846 14.14918119

Ni 2.34689055 4.06195580 14.16011618

Ni -2.58226370 4.82197689 5.27973946

Ni 0.36983110 4.80093032 5.29356345

Ni 2.15462288 0.56825721 9.72315432

Ni 5.09497677 0.56585056 9.71794332

Ni 0.89480367 1.46225173 14.16116918

Ni 3.83764288 1.48335498 14.14739919

H 3.57719530 1.33234950 7.34872146

H 0.63882659 1.48201509 7.40012944

H -1.03731719 3.91095547 7.63756732

H 1.87874776 3.90688427 7.64021332

H -2.32696771 4.80350703 12.04086020

H 0.63077923 4.95308502 12.09199818

H -0.65014085 2.37524690 11.80374632

H 2.32875198 2.37936449 11.80056032

H 1.05299296 3.42085501 16.16972521

H 3.15420396 2.86359005 3.27123742

O 2.35128290 0.60835081 15.02786876

O -0.73959715 2.35349948 10.76497582

O 2.22246257 2.35329849 10.76195182

O 3.90486772 3.11378339 15.10784273

O 0.96352717 3.29600313 15.19912968

O 3.24381464 2.98825641 4.24185996

O 0.30257906 3.17053799 4.33301191

O 4.89852636 0.52253608 4.41303988

O 1.94428840 0.53815609 4.41957387

O 0.69386200 1.45877832 8.84762574

O 3.64112131 1.44358605 8.81079776

O -0.57072496 2.34309472 13.28391260

O 2.37396515 2.35763767 13.28331860

O 3.47597760 1.32603140 6.28729797

O 0.52826234 1.44623490 6.35147694

O 1.83352252 3.92670432 6.15759004

O -1.11671378 3.94109782 6.15696904

O -0.94811469 3.93416131 8.67625682

O -2.38253358 4.82850111 10.59325590

O 0.56722146 4.84394076 10.63013788

O 1.98482176 3.93392426 8.67887582

O 5.30545696 0.59267926 15.02144277

O -2.21575051 4.83808132 13.08943170

O 0.73156142 4.95840335 13.15358367

***OH surface with Fe(HS)**

Supercell lattice vectors

5.89489800, 0.00000000, 0.00000000

-3.04229787, 5.15339025, 0.00000000

0.00000000, 0.00000000, 26.99998900

Atomic positions

Fe -0.99693838 1.95705664 5.23275987

Fe 2.11860404 4.20191466 14.17947622

Ni 1.95079066 1.98139610 5.23205787

Ni 0.56642556 3.07940835 9.70514605

Ni 3.51398600 3.07959902 9.70644205

Ni -0.82911146 4.17860072 14.18274322

Ni -2.52458690 4.59382999 5.20924288

Ni 0.42298373 4.59234581 5.21475088

Ni 2.08772298 0.50201236 9.70601005

Ni 5.03489149 0.50187837 9.70614505

Ni 0.69945421 1.56774377 14.20013121

Ni 3.64746201 1.56604830 14.20420821

H 3.47266315 1.28694069 7.38433499

H 0.60094275 1.26309080 7.35903600

H -1.03404345 3.80284127 7.60090190

H 1.92058393 3.78387164 7.56296692

H -2.34101667 4.87198938 12.03138410

H 0.53029312 4.89877155 12.05665609

H -0.79171042 2.37742899 11.84708217

H 2.16317425 2.35424904 11.80885019

H 3.75238924 3.58331200 16.25159038

H 0.63691449 3.43328650 16.29182036

H 3.25633143 2.56876406 3.16402071

H 0.47561146 2.73095156 3.11938973

O 2.18057339 0.65655738 15.07174486

O -0.83657470 2.28541005 10.80890560

O 2.11353387 2.31994807 10.75479762

O 3.64284268 3.40964790 15.29392777

O 0.66762180 3.35717608 15.31766076

O 3.36426285 2.74802989 4.12044132

O 0.44846382 2.80518614 4.09373833

O 4.93338760 0.22016829 4.29235025

O 1.98518665 0.34653973 4.34421723

O 0.61976984 1.30815720 8.79862142

O 3.56036247 1.40123258 8.83650240

O -0.78689047 2.49268456 13.33567257

O 2.17411303 2.38271121 13.29376858

O 3.44418979 1.19619464 6.34132542

O 0.53346238 1.23002149 6.30406543

O 1.90888109 3.67008994 6.07826452

O -1.04983464 3.77106546 6.12100551

O -0.98169803 3.83814199 8.65635847

O -2.42699772 4.75786240 10.57659769

O 0.51276478 4.84879912 10.61272368

O 1.96965345 3.87353032 8.60235850

O 5.12505633 0.79336958 15.12390884

O -2.31673594 4.96200880 13.07452867

O 0.59625550 4.93280454 13.11054666

***OH surface with Fe(LS)**

Supercell lattice vectors

5.89489800, 0.00000000, 0.00000000

-3.04229787, 5.15339025, 0.00000000

0.00000000, 0.00000000, 26.99998900

Atomic positions

Fe -1.02273062 1.96629666 5.28054985

Fe 2.14621965 4.19145327 14.13287424

Ni 1.92693592 1.97312491 5.28343885

Ni 0.56466479 3.08139240 9.70660405

Ni 3.51211075 3.08139755 9.70668505

Ni -0.80340848 4.18461988 14.13001224

Ni -2.54830432 4.59459269 5.25479186

Ni 0.39913957 4.59086679 5.25549386

Ni 2.08578026 0.50475396 9.70660405

Ni 5.03326159 0.50475912 9.70663105

Ni 0.72436848 1.56681101 14.15793023

Ni 3.67180495 1.56316756 14.15860523

H 3.52607253 1.31856189 7.37329200

H 0.50059264 1.35396053 7.35517500

H -1.08480150 3.77866156 7.60527590

H 1.86958099 3.75634223 7.60829990

H -2.40019010 4.84078560 12.04015909

H 0.62522308 4.80612905 12.05784409

H -0.74193629 2.40478318 11.80517819

H 2.21218360 2.38247931 11.80817519

H 3.62108299 3.80352667 16.13041443

H 0.78493423 3.64601845 16.17261541

H 3.39615517 2.35146621 3.28373866

H 0.33715205 2.51267971 3.24043068

O 2.20525599 0.66725066 15.00432589

O -0.79328662 2.30532790 10.76845961

O 2.15672784 2.30206065 10.76473361

O 3.59561346 3.48087291 15.20417981

O 0.73980134 3.43663620 15.21643780

O 3.42234910 2.67607826 4.20927129

O 0.38318721 2.72140232 4.19666229

O 4.90884327 0.31662430 4.39063021

O 1.96058796 0.33705234 4.40915220

O 0.62325653 1.39656361 8.83234440

O 3.57892550 1.38826149 8.84222640

O -0.76043412 2.48515546 13.31342458

O 2.19454784 2.46485625 13.29757558

O 3.46878999 1.23955011 6.33257742

O 0.43333965 1.26449767 6.31419043

O 1.88404258 3.67267178 6.09997251

O -1.07111281 3.69294522 6.11584851

O -1.02759185 3.86067261 8.64844748

O -2.44961113 4.77454907 10.57108969

O 0.50599547 4.76627273 10.58075569

O 1.92251751 3.85737444 8.64472148

O 5.15192169 0.68770962 15.02290188

O -2.34474647 4.91801946 13.08119767

O 0.69065218 4.89363877 13.09917966

***OH surface for pure NiOOH**

Supercell lattice vectors

5.89493800, 0.00000000, 0.00000000

-3.04231207, 5.15341903, 0.00000000

0.00000000, 0.00000000, 26.99998700

Atomic positions

Ni -1.10728701 2.01071466 5.26362047

Ni 1.84018199 2.01071466 5.26362047

Ni 0.56611848 3.08227538 9.70657633

Ni 3.51358748 3.08227538 9.70657633

Ni -0.71813924 4.14609052 14.14980219

Ni 2.22932976 4.14609052 14.14980219

Ni -2.63640073 4.62711580 5.24207448

Ni 0.31106523 4.62712096 5.24207448

Ni 2.08724467 0.50558648 9.70649533

Ni 5.03471367 0.50558648 9.70649533

Ni 0.81099462 1.52955538 14.17140218

Ni 3.75846362 1.52955538 14.17140218

H 3.51383616 1.33717310 7.36160046

H 0.56636127 1.33717310 7.36160046

H -1.11856997 3.77850229 7.60611234

H 1.82889313 3.77850229 7.60611234

H -2.38806139 4.82280659 12.05155220

H 0.55941654 4.82280143 12.05155220

H -0.69958321 2.38349753 11.80736431

H 2.24787989 2.38349753 11.80736431

H -1.97824279 3.59348424 16.19845320

H 0.96922545 3.59352547 16.19845320

H 3.09877090 2.56314056 3.21496945

H 0.15129582 2.56315087 3.21494245

O 2.27635676 0.64238399 15.02746376

O -0.78762259 2.30477905 10.77088881

O 2.15984641 2.30477905 10.77088881

O 3.81378934 3.37331988 15.24886366

O 0.86632320 3.37332503 15.24889066

O 3.20278046 2.78323278 4.16442399

O 0.25530841 2.78323794 4.16442399

O 4.83541990 0.36084240 4.38620189

O 1.88794500 0.36084240 4.38620189

O 0.63143222 1.39436574 8.84003874

O 3.57890122 1.39436574 8.84003874

O -0.65693397 2.44022637 13.31723059

O 2.29052914 2.44022637 13.31723059

O 3.41615983 1.25710442 6.32466595

O 0.46869083 1.25710442 6.32466595

O 1.77914082 3.71663034 6.09621906

O -1.16832818 3.71663034 6.09621906

O -1.02771004 3.85963257 8.64207484

O -2.44678842 4.77020564 10.57297891

O 0.50068647 4.77020564 10.57297891

O 1.91975611 3.85962742 8.64204784

O 5.22382576 0.64238399 15.02746376

O -2.29353024 4.90000480 13.08905370

O 0.65393876 4.90000480 13.08905370

***OOH surface with Fe(HS)**

Supercell lattice vectors

5.89489800, 0.00000000, 0.00000000

-3.04229787, 5.15339025, 0.00000000

0.00000000, 0.00000000, 26.99998900

Atomic positions

Fe -0.99513745 2.08789091 5.32496483

Fe 2.19744458 4.22069361 14.18687422

Ni 1.96619567 2.09036453 5.23500087

Ni 0.57343936 3.14352683 9.71246304

Ni 3.51893952 3.14547996 9.70020505

Ni -0.73207084 4.17738452 14.13157824

Ni -2.50409198 4.71050274 5.20886488

Ni 0.44280769 4.74114995 5.19498688

Ni 2.09374533 0.56683170 9.71578404

Ni 5.03901300 0.56620299 9.70444405

Ni 0.79009685 1.58120958 14.19141022

Ni 3.74621236 1.56113712 14.20763721

H 3.42374318 1.38645265 7.40898598

H 0.57728396 1.35883563 7.40477398

H -1.04987570 3.92895504 7.60673390

H 1.86448670 3.84656263 7.59917390

H -2.33203540 4.92977950 12.01812710

H 0.57941488 4.88934600 12.02657810

H -0.72831386 2.43636831 11.80844519

H 2.26708855 2.38376766 11.81160419

H 0.61152477 3.39471337 16.26290337

H 3.40665161 2.81344703 3.15718971

H -1.53300843 4.88669715 16.42700931

H -0.06974005 1.33753667 3.03930776

O 2.26039966 0.64884275 15.07255486

O -0.78830183 2.36470526 10.76538161

O 2.17470573 2.35675358 10.77024161

O 3.80600449 3.49853357 15.29174077

O 0.77111255 3.34693114 15.29614177

O 3.46815659 2.91334550 4.13037732

O 0.55024556 2.76784983 4.10694133

O 4.97710943 0.38931287 4.27331526

O 2.00184341 0.45011256 4.34975223

O 0.63991368 1.45351372 8.85013739

O 3.57864780 1.45059175 8.83118340

O -0.71158097 2.48183667 13.30416358

O 2.28606901 2.40576748 13.32314457

O 3.38541424 1.34114920 6.34853441

O 0.48801150 1.29638685 6.36500441

O 1.89680327 3.80360397 6.05874353

O -0.99988513 3.91043890 6.11779251

O -1.01609280 3.93748390 8.65322647

O -2.44550692 4.83484889 10.57076569

O 0.51125232 4.83296791 10.58596669

O 1.92441625 3.92171968 8.63635148

O 5.22293332 0.80212004 15.11005784

O -2.26046187 4.99284153 13.05870668

O 0.67030829 4.93995229 13.08141367

O -1.84780010 3.94287950 16.63466622

O 0.41275805 2.20483164 2.80659486

***OOH surface with Fe(LS)**

Supercell lattice vectors

5.89489800, 0.00000000, 0.00000000

-3.04229787, 5.15339025, 0.00000000

0.00000000, 0.00000000, 26.99998900

Atomic positions

Fe -0.95265523 2.04894158 5.29882884

Fe 2.19688957 4.24764584 14.14947924

Ni 1.98927267 2.03920168 5.27163985

Ni 0.58064457 3.14199627 9.71238204

Ni 3.52684464 3.14259406 9.70487605

Ni -0.75504305 4.22461534 14.11834825

Ni -2.48257074 4.65739190 5.25508886

Ni 0.45916206 4.68627665 5.24777186

Ni 2.10171345 0.56532691 9.71192304

Ni 5.04665541 0.56525992 9.70663105

Ni 0.76627542 1.62461658 14.16130523

Ni 3.71635238 1.59857650 14.16859523

H 3.53215714 1.40225810 7.36991700

H 0.51772546 1.39579575 7.37558700

H -1.03541178 3.85076264 7.61151290

H 1.88606810 3.82076991 7.60800290

H -2.38536074 4.90223978 12.03991609

H 0.65022163 4.86579500 12.06324409

H -0.72387992 2.45409082 11.80620419

H 2.24995984 2.43827506 11.81038919

H 0.86361393 3.61460338 16.19003040

H 3.58243907 2.63656206 3.24853068

H -1.74578969 4.95134128 16.38119033

H -0.06431717 1.30248847 3.05453576

O 2.22305373 0.69906254 15.02287488

O -0.77990937 2.36544735 10.76929661

O 2.17601784 2.36137102 10.77016061

O 3.65976794 3.57293822 15.19664681

O 0.80185624 3.47238527 15.22029880

O 3.53107531 2.79838882 4.21618328

O 0.46659377 2.69643930 4.19882229

O 4.98076453 0.40803513 4.36911122

O 2.01524296 0.39628025 4.41244620

O 0.63814338 1.45521434 8.84379240

O 3.59149794 1.45111224 8.83620540

O -0.72483725 2.52468196 13.31277658

O 2.25696487 2.51488021 13.31582758

O 3.50053379 1.33228537 6.32274942

O 0.46445758 1.30924456 6.33619542

O 1.92705367 3.74959644 6.07632052

O -0.97821693 3.77583750 6.12324651

O -1.00754822 3.92515699 8.65460347

O -2.43793548 4.83295760 10.57176469

O 0.52467008 4.82907194 10.58480569

O 1.93520027 3.91663328 8.64156248

O 5.19100756 0.74809705 15.02165988

O -2.31183216 4.97870578 13.07828167

O 0.72306378 4.94320408 13.10563266

O -2.11678487 4.02794651 16.55493526

O 0.33940681 2.21177326 2.85565384

***OOH surface for pure NiOOH**

Supercell lattice vectors

5.89493800, 0.00000000, 0.00000000

-3.04231207, 5.15341903, 0.00000000

0.00000000, 0.00000000, 26.99998700

Atomic positions

Ni -1.04048511 2.10774323 5.28759645

Ni 1.90850501 2.09619957 5.26510546

Ni 0.60403788 3.13847857 9.71289432

Ni 3.55133821 3.13867440 9.70679233

Ni -0.69693276 4.16918299 14.13090220

Ni 2.24918265 4.18091217 14.15471618

Ni -2.56237976 4.71289446 5.24013048

Ni 0.38090826 4.73875947 5.23230048

Ni 2.12514202 0.56218648 9.71194932

Ni 5.07255071 0.56161961 9.70779133

Ni 0.82490009 1.56418636 14.17915117

Ni 3.77679784 1.53815128 14.18684617

H 3.55645369 1.40299256 7.38090545

H 0.62760064 1.40973324 7.37353445

H -1.05597807 3.86181247 7.61302433

H 1.86698455 3.84569772 7.61294333

H -2.36630760 4.86724967 12.04561220

H 0.60064409 4.87382028 12.03786320

H -0.68305351 2.41487154 11.80660832

H 2.28920846 2.43142948 11.80671632

H 0.95456399 3.56890243 16.19993820

H 3.20080805 2.70702918 3.21939745

H -1.58977332 4.89623765 16.40959310

H -0.13764543 1.38446087 3.00415355

O 2.27498212 0.66358515 15.04995475

O -0.75580049 2.35644208 10.77118581

O 2.19697810 2.35974027 10.77558681

O -2.05847010 3.48859155 15.26360565

O 0.87923305 3.39687100 15.23563366

O 3.27790409 2.87988516 4.18345899

O 0.32178062 2.79007652 4.15645900

O 4.92514179 0.45992719 4.36894890

O 1.95648312 0.45513966 4.38614789

O 0.66794033 1.45230048 8.84241474

O 3.61262662 1.45007420 8.83758174

O -0.65975477 2.44887896 13.32560058

O 2.32744723 2.47332162 13.34136858

O 3.47490280 1.34631526 6.33311695

O 0.53567322 1.33823985 6.33260395

O 1.83025888 3.80390865 6.07837207

O -1.07788636 3.82784628 6.09349207

O -0.98376979 3.92068512 8.64841984

O -2.40760834 4.82485249 10.57724491

O 0.54287802 4.82714061 10.58210490

O 1.95847120 3.91743847 8.64418084

O 5.24322606 0.66864581 15.03299876

O -2.27335579 4.93881520 13.08654270

O 0.68280253 4.93031721 13.08584070

O -1.88658936 3.94716339 16.60377701

O 0.15323831 2.33556558 2.81445164

References

[1] Hall, D. S., Lockwood, D. J., Bock, C. & MacDougall, B. R. Nickel hydroxides and related materials: a review of their structures, synthesis and properties. *Proceedings of the Royal Society A: Mathematical, Physical and Engineering Sciences* **471**, 20140792 (2015).

[2] Martirez, J. M. P. & Carter, E. A. Effects of the aqueous environment on the stability and chemistry of β-NiOOH surfaces. *Chemistry of Materials* **30**, 5205–5219 (2018).

[3] Casas-Cabanas, M., Canales-Vázquez, J., Rodríguez-Carvajal, J. & Palacín, M. R. Deciphering the structural transformations during nickel oxyhydroxide electrode operation. *Journal of the American Chemical Society* **129**, 5840–5842 (2007).

[4] Majzlan, J., Grevel, K.-D. & Navrotsky, A. Thermodynamics of Fe oxides: Part ii. enthalpies of formation and relative stability of goethite (α-FeOOH), lepidocrocite (γ-FeOOH), and maghemite (γ-Fe_2O_3). *American Mineralogist* **88**, 855–859 (2003).

[5] Kazimirov, V. Y. *et al.* Atomic structure and lattice dynamics of Ni and Mg hydroxides. *Solid State Ionics* **181**, 1764–1770 (2010).

[6] Zaffran, J. & Caspary Toroker, M. Benchmarking density functional theory based methods to model NiOOH material properties: Hubbard and van der waals corrections vs hybrid functionals. *Journal of Chemical Theory and Computation* **12**, 3807–3812 (2016).

[7] Natta, G. Constitution of hydroxides and hydrates. *Gazzetta Chimica Italiana* **58**, 344–358 (1928).

[8] Cairns, R. & Ott, E. X-ray studies of the system nickel—oxygen—water. i. nickelous oxide and hydroxide. *Journal of the American Chemical Society* **55**, 527–533 (1933).

[9] McEwen, R. Crystallographic studies on nickel hydroxide and the higher nickel oxides. *The Journal of Physical Chemistry* **75**, 1782–1789 (1971).

[10] Glemser, O. & Einerhand, J. Die struktur höherer nickelhydroxyde. *Zeitschrift für anorganische Chemie* **261**, 43–51 (1950).

[11] Takada, T., Bando, Y., Kiyama, M., Miyamoto, H. & Sato, T. The magnetic property of $Ni(OH)_2$. *Journal of the Physical Society of Japan* **21**, 2745–2746 (1966).

[12] Szytula, A., Murasik, A. & Balanda, M. Neutron diffraction study of $Ni(OH)_2$. *Physica Status Solidi (b)* **43**, 125–128 (1971).

[13] Friebel, D. *et al.* Identification of highly active Fe sites in (Ni, Fe)OOH for electrocatalytic water splitting. *Journal of the American Chemical Society* **137**, 1305–1313 (2015).
